# Efficacy of *Lactobacillus rhamnosus* and Its Metabolites to Mitigate the Risk of Foodborne Pathogens in Hydroponic Nutrient Solution

**DOI:** 10.3390/microorganisms13081858

**Published:** 2025-08-08

**Authors:** Esther Oginni, Robin Choudhury, Veerachandra Yemmireddy

**Affiliations:** 1 School of Earth, Environmental, and Marine Sciences, University of Texas Rio Grande Valley, 1201 W University Dr, Edinburg, TX 78539, USA; 2 School of Integrative Biological and Chemical Sciences, University of Texas Rio Grande Valley, 1201 W University Dr, Edinburg, TX 78539, USA

**Keywords:** biological intervention, biofilm inhibition, antimicrobial activity, microbial safety, controlled environmental agriculture

## Abstract

Hydroponic nutrient solution (HNS) has been established as an ideal conduit for pathogen contamination and proliferation. This study evaluated the efficacy of lactic acid bacteria and their metabolites in mitigating the risk of foodborne pathogens in HNS when compared to conventional chemical treatments. Hoagland’s HNS were prepared according to the manufacturer’s instructions and inoculated with *Salmonella* Typhimurium, *Escherichia coli* 0157:H7, and *Listeria innocua* at 10^5^ CFU/mL cell concentration. These nutrient solutions were subjected to treatment with various concentrations of *Lactobacillus rhamnosus* live cells, a cell-free extract (CFE) of *L. rhamnosus* metabolites, sodium hypochlorite and peroxyacetic acid at 22 ± 1 °C for up to 96 h using appropriate controls. The survived cells were enumerated on respective selective media at regular intervals. Additionally, the impact of these treatments on lettuce growth and the physico-chemical properties of HNS, such as pH, electrical conductivity, salinity, total dissolved solids, and % lactic acid content, were determined over 21 days using standard procedures. Both *S.* Typhimurium and *E. coli* O157: H7, when in combination with *L. rhamnosus,* remained stable in HNS over a 96 h period, while *L. innocua* showed a 3-log reduction. Whereas CFE treatment of HNS showed a significant reduction in *Salmonella* and *E. coli* O157: H7 (both undetectable after 96 h; LOD: <1 log CFU/mL). Interestingly, *L. innocua* levels remained stable after CFE treatment. PAA treatments at 12 mg/L notably reduced *Salmonella* and *L. innocua* growth, but not *E. coli* O157:H7. Lettuce plants in untreated control were significantly taller and heavier compared to those treated with CFE. These findings highlight the potential of biological interventions while emphasizing their limitations in hydroponic systems for pathogen control.

## 1. Introduction

Agricultural food production faces substantial challenges from rapid urbanization, climate change, food safety concerns, and food insecurity [1,2,3]. Controlled environment agriculture offers promising solutions by optimizing factors such as temperature, light, nutrients, and relative humidity to enhance crop productivity [3]. Among these techniques, hydroponic systems have gained significant attention due to their advantages, including efficient land and water use, precise nutrient regulation, reduced environmental footprints, and consistent food production [2,4,5]. In hydroponic systems, nutrient solutions provide essential elements in ionic form, with availability influenced by factors like electrical conductivity and pH [6]. Although these systems reduce many external biotic and abiotic stressors, planting inputs such as irrigation water can serve as pathways for pathogen contamination. High nutrient concentrations and optimal moisture levels in hydroponic nutrient solutions may support pathogen survival, proliferation, and internalization into plant systems [4,7,8,9].

Pathogen contamination in hydroponically grown lettuce, spinach, basil, and tomatoes has been documented [9,10]. For example, an *E. coli* O104:H4 outbreak in 2011 was linked to hydroponically grown fenugreek sprouts, and recent *Salmonella* Typhimurium outbreaks were associated with hydroponically grown leafy greens from similar setups [7,11]. Key contamination pathways, such as seeds, growth media, nutrient solutions, and tools and equipment highlight the need for effective antimicrobial interventions to mitigate risks [6]. Various chemical and physical interventions, including ultraviolet radiation, heat treatments, ozone, sodium hypochlorite, and peracetic acid have been employed to disinfect nutrient solutions [8,12,13]. However, these methods often present limitations related to costs, residue buildup, and potential adverse effects on plant health [4,14]. Thus, there is a need for sustainable alternatives that preserve crop performance and effectively reduce microbial risks.

Lactic acid bacteria (LAB) are Gram-positive, non-spore-forming bacteria known for their antagonistic activities against pathogenic bacteria due to their ability to produce antimicrobial compounds such as lactic acid, hydrogen peroxide, bacteriocins, and other organic acids [15,16]. These metabolites can inhibit pathogenic bacteria by reducing pH and disrupting cellular functions [17]. Bacteriocins produced by LAB are able to kill targeted cells and can be applied either directly as a purified compound, as a crude bacterial metabolite, or as a bacteriocin-producing organism [18]. LABs are generally regarded as safe and have been widely used in the food industry. Studies have reported LAB’s ability to inhibit the growth of spoilage and pathogenic bacteria on animal [19,20,21,22] and plant [23,24]. For example, Iglesias et al. [25] evaluated the antagonistic activities of *L. rhamnosus* and *L. acidophilus* against *Salmonella* and *Listeria monocytogenes* in minimally processed pears at different storage temperatures. They observed that co-inoculation of the pathogens with *L. rhamnosus* significantly reduced *Salmonella* and *L. monocytogenes* populations, while co-inoculation of the pathogens with *L. acidophilus* increased the pathogen populations [25] Although LAB has been extensively studied for its antagonistic activities against pathogens in food systems, its application in hydroponic systems remains less explored. Therefore, this study aimed to evaluate the efficacy of *Lactobacillus rhamnosus* and its metabolites against foodborne pathogens in a hydroponic nutrient solution (HNS) and to assess the impacts of these treatments on plant growth performance.

## 2. Materials and Methods

### 2.1. Selection of Bacterial Strains and Inoculum Preparation

Three bacterial pathogens/surrogates *Salmonella* Typhimurium (ATCC 14028), *Escherichia coli* O157:H7 (ATCC 35150), and *Listeria innocua* (ATCC 15742), were evaluated alongside *Lactobacillus rhamnosus* (ATCC 53103). Stock cultures were maintained at −70 °C in tryptic soy broth (TSB; Bacto^TM^, Becton Dickinson, Sparks, MD, USA) or DeMan Rogosa and Sharpe (MRS) agar for *L. rhamnosus* with 25% glycerol. The cultures were revived by streaking 10 µL of stock onto Xylose Lysine Deoxycholate (XLD; *S*. Typhimurium), Sorbitol MacConkey (SMAC; *E. coli* O157:H7), Oxford (*L. innocua*), and MRS (*L. rhamnosus*) agar plates, respectively, and incubated in a CO_2_ incubator at 37 °C for 24 to 48 h. Afterwards, individual cultures of each bacterium were activated by two successive passages by first inoculating 10 µL of pure culture either in 10 mL of TSB or MRS (for *L. rhamnosus*) broth and incubated aerobically at 37 °C for 24 or 48 h in a shaker incubator (New Brunswick Scientific^TM^, Model E24 Incubator, Enfield, CT, USA); and this procedure was repeated for a second reactivation. After incubation, the cells were harvested by centrifugation (Model 5920R, Eppendorf™, Hamburg, Germany) at 4 °C, 4000× *g* for 20 min. The supernatants were decanted, and the cells were resuspended in 10 mL of sterile Milli-Q™ (Model IX 7003, Millipore Sigma, Burlington, MA, USA) water. Sterile water was employed as a dilutant to prevent the introduction of additional nutrient sources into the HNS [26]. Cell concentrations were adjusted to 10^5^ CFU/mL and verified by plating on selective media and incubating at 37 °C for 24 ± 2 h (test organisms) or 48 ± 2 h (*L. rhamnosus*).

### 2.2. Preparation, Inoculation, and Treatment of Hydroponic Nutrient Solutions

Sterile Hoagland’s No. 2 basal salts inorganic hydroponic nutrient solution was prepared following the manufacturer’s instructions (Caisson Labs Inc., Smithfield, UT, USA).

#### 2.2.1. Treatment of Pathogens with *L. rhamnosus*

Preliminary experiments were conducted using different volumes (10, 20, and 30 mL) of HNS consisting of varying proportions (1:1, 1:3, and 3:1 (*v*/*v*)) of *S.* Typhimurium to *L. rhamnosus*. Results indicated a 30 mL reaction mixture volume as optimal for multiple samplings and no significant difference in survival was observed with an increasing proportion of *L*. *rhamnosus* from 1 to 3 parts in the mixture. Hence, the following treatments were tested: (i) Control: 27 mL HNS + 3 mL pathogen or *L. rhamnosus*, (ii) Treatment-1: 24 mL HNS + 3 mL pathogen + 3 mL *L. rhamnosus*, and (iii) Treatment-2: 26 mL HNS + 3 mL pathogen + 1 mL *L. rhamnosus*. Following inoculation of the HNS with selected bacterial pathogens/surrogate, lactic acid bacteria was inoculated into the 50 mL centrifuge tube containing the treatments. The reaction mixtures were vortexed for 1 min and stored at 23 ± 2 °C. Experiments were conducted separately for each pathogen by following methods described in Laury-Shaw et al. [15] and Iglesias et al. [25].

#### 2.2.2. Treatment of Pathogens with Cell-Free Extracts of *L. rhamnosus*

During preliminary studies, the cell-free extract (CFE) of *L. rhamnosus* inhibited *S.* Typhimurium. The methodology for the preparation and application of the CFE was adopted by following Kohestani et al. [27]. The procedure for the preparation of the CFE involves, a 10 µL culture of *L. rhamnosus* was inoculated into 10 mL of sterile MRS broth and incubated at 37 °C for 48 ± 2 h. Following incubation, the cells were harvested by centrifugation at 4000× *g* for 20 min at 4 °C and the supernatant was filtered through a 0.22 µm pore-size filter twice to remove any residual bacterial cells and large particles. Absence of viable *L. rhamnosus* was confirmed by plating onto MRS agar. The resulting CFE was collected in sterile centrifuge tubes and either used immediately in experiments or stored at 4 °C for no longer than 24 h. To evaluate the efficacy of the CFE, reaction mixtures (30 mL total), included HNS, pathogen inoculum, and CFE in proportions: (i) Treatment-3: 24 mL HNS + 3 mL pathogen + 3 mL CFE; (ii) Treatment-4: 26 mL HNS + 3 mL pathogen + 1 mL CFE. Each mixture was vortexed for 1 min and stored at 23 ± 2 °C under static conditions to replicate HNS storage conditions. Separate experiments were conducted for each test bacterium.

#### 2.2.3. Treatment with Conventional Sanitizers

To compare the efficacy of conventional sanitizers with the above-described biological interventions, HNS containing test pathogens was treated with sodium hypochlorite (NaOCl, 5% available chlorine, Ricca Chemical Company, Arlington, TX, USA) and peracetic acid (PAA; SaniDate^®^ 15, Biosafe Systems, East Hartford, CT, USA) at 4 and 12 mg/L. Free chlorine concentration was determined with a colorimetric method by following DPD-free chlorine assays using a DR900 portable colorimeter (HACH^®^, Loveland, CO, USA) and PAA concentrations were determined by using MQuant^®^ (Millipore Sigma, Burlington, MA, USA) test strips. Preliminary data showed no survivor at 4 mg/L NaOCl; therefore, only PAA treatments were evaluated: (i) Treatment-5: 27 mL HNS with 4 mg/L PAA + 3 mL pathogen; (ii) Treatment-6: 27 mL HNS with 12 mg/L PAA + 3 mL pathogen. After inoculating the PAA-treated HNS with the test bacteria separately in a 50 mL sterile centrifuge tubes, the reaction mixtures were vortexed for 1 min and stored at 23 ± 2 °C.

### 2.3. Measurement of Antimicrobial Efficacy of Different Treatments

Following the respective treatments, the viability of *S.* Typhimurium, *E. coli* O157:H7, *L. innocua* and *L. rhamnosus* were determined by collecting 1 mL aliquots (at 0, 6, 12, 24, 48, 72, and 96 h) into 9 mL phosphate-buffer saline (PBS). The collected samples were 10-fold serially diluted and plated on selective media for each organism. Plates were incubated at 37 °C for 24 ± 2 h (for pathogens) or 48 ± 2 h (for *L. rhamnosus*) and the survivors were enumerated as CFU/mL.

### 2.4. Seedling Gerination and Masurement

Lettuce seeds (*Lactuca sativa*) of Butterscotch variety were germinated under sterile conditions using a Petri dish containing dampened filter paper at 25 °C in the dark for 7 days. During this period, the seedlings were regularly watered to prevent wilting. When the seedlings reached approximately 3 cm tall and developed their first leaves, they were transferred to 50 mL Falcon tubes wrapped with aluminum foil to maintain darkness around the root region. The tubes were filled with the respective treatment nutrient solutions and were placed in a growth chamber (Thermo Scientific PR505755L Precision Incubator, 17.79 L, Waltham, MA, USA) set to a photo period of 16 h light at 21 °C and 8 h dark at 19 °C for 21 days following the method described in Xylia et al. [28]. Plant height and weight were measured every 3 days using a digital caliper (VINCA DCLA-0605, Valencia, CA, USA) and an analytical balance (Mettler Toledo ME104TE/00, Allendale, MI, USA). At the conclusion of the experiment, plant dry weight was determined after oven drying at 65 °C for 72 h.

### 2.5. Measurement of Physicochemical Properties

To determine the consistency of the HNS over the experimental period, changes in temperature, pH, electrical conductivity (EC), total dissolved solids (TDS), and % lactic acid (for CFE treatments) were recorded at day 0 and every 3 days up to 21 days. The temperature and pH were analyzed using a pH meter (Model A211, Orion™, Waltham, MA, USA). The EC, TDS, and salinity of the samples were analyzed using a conductivity meter (Thermo Scientific™, Eutech, Singapore). The titratable acidity was determined by titrating a known volume of CFE with 0.1N NaOH using phenolphthalein as an indicator, and expressed as % lactic acid.

### 2.6. Experimental Design and Data Analysis

A completely randomized design was used with three replicates (n = 3) per treatment. Duplicate samples were analyzed at each time point. Survival data (CFU/mL) for each organism in the treatments were log-transformed (log CFU/mL) before statistical analysis. The data were analyzed by one-way analysis of variance (ANOVA) to compare the mean log survival CFU/mL at each sampling time point across different treatment groups. Pairwise comparisons of the means were performed with Tukey–Kramer post hoc test at a significance level of 0.05 (JMP^®^PRO 16, SAS Institute, Inc., Cary, NC, USA).

## 3. Results

### 3.1. Effect of Treatments on Test Organisms

#### 3.1.1. Kinetics of Tested Bacteria in Hydroponic Nutrient Solution

**Figure 1:**
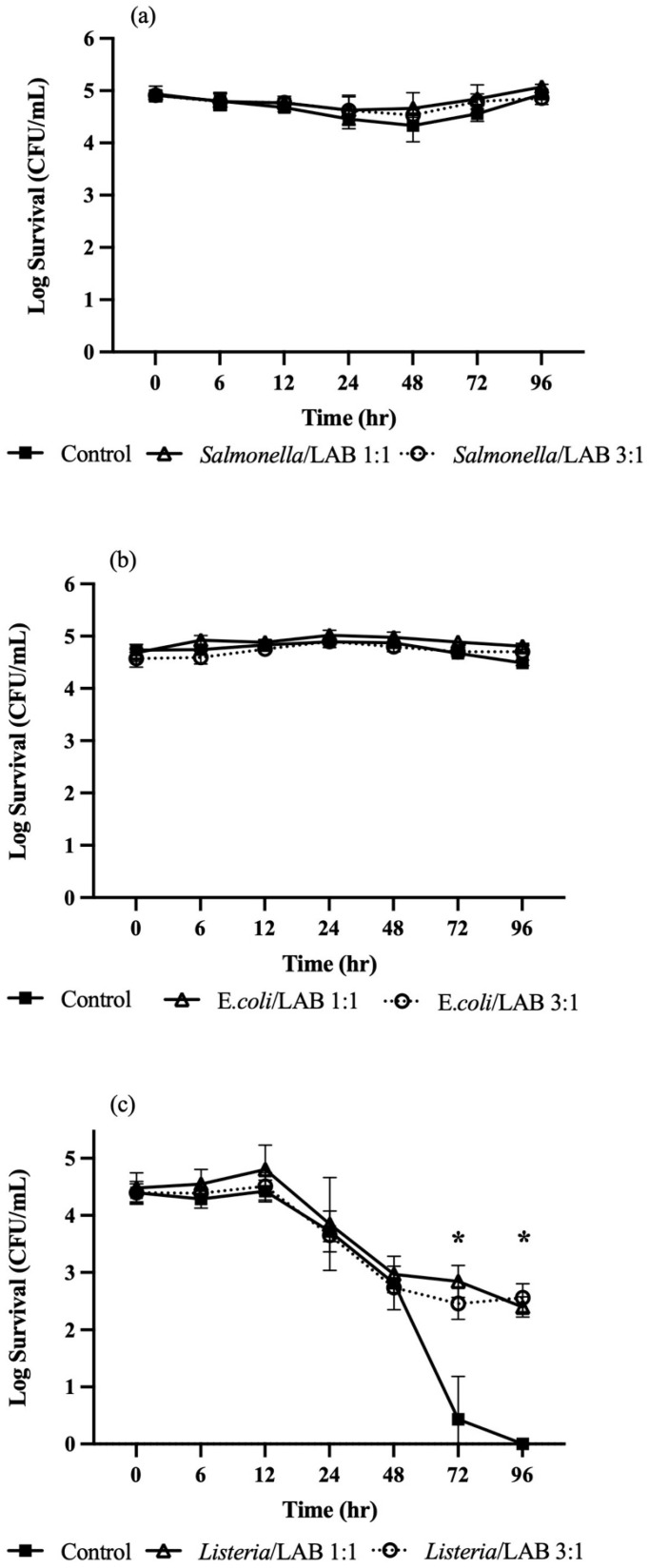
Survival kinetics of *Salmonella* Typhimurium (**a**), *E. coli* O157:H7 (**b**), and *Listeria innocua* (**c**) in *Lactobacillus rhamnosus*-treated hydroponic nutrient solution. Means with an asterisk indicate significant difference (*p* ≤ 0.05) between treatments.

Figure 1 shows the survival kinetics of *S.* Typhimurium, *E. coli* O157:H7, and *L. innocua* in HNS with or without *L. rhamnosus*. No significant effect (*p* > 0.05) of *L. rhamnosus* on *S.* Typhimurium was observed when compared to control (Figure 1a). *Salmonella* counts in control were decreased from 4.91 to 4.34 log by 48 h but recovered to 4.93 log by 96 h. A similar trend was observed in *L. rhamnosus*-treated HNS regardless of the treatment ratio (1:1 or 3:1 *v*/*v*). No significant differences (*p* > 0.05) in the levels of *E. coli* O157:H7 were observed between control and treated samples, with CFU reductions remaining <0.5 log over 96 h (Figure 1b). Interestingly, *L. innocua* showed a 4.4 log CFU/mL decline in the control, but only 2 log reduction in *L. rhamnosus*-treated HNS (Figure 1c).

**Figure 2:**
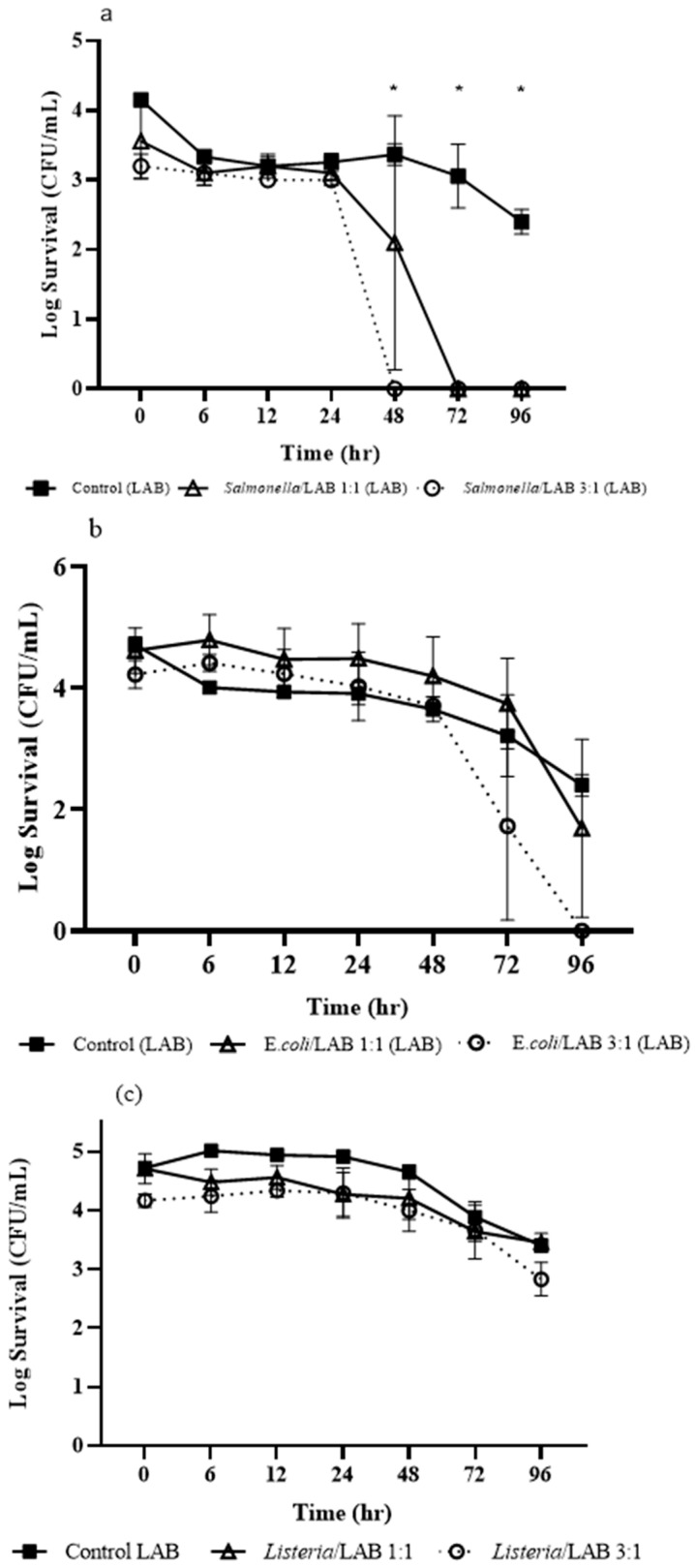
Survival kinetics of *Lactobacillus rhamnosus* in hydroponic nutrient solution containing *Salmonella* Typhimurium (**a**), *E. coli* O157:H7 (**b**), and *Listeria innocua* (**c**). Means with an asterisk indicate significant difference (*p* ≤ 0.05) between treatments.

Figure 2a–c shows *L. rhamnosus* survival in HNS containing different pathogens. *L. rhamnosus* populations declined within 24 h in all conditions. With *S*. Typhimurium at 3:1 ratio (i.e., 3 parts *Salmonella* and 1 part *L. rhamnosus*), *L. rhamnosus* was undetectable after 48 h; at a 1:1 ratio, *L. rhamnosus* persisted up to 72 h (Figure 2a). Co-inoculation with *E. coli* O157:H7 led to slight changes over 48 h, followed by sharper decline afterward (Figure 2b). At 96 h, *L. rhamnosus* declined to 1.69 log CFU/mL at 1:1 ratio treatment and undetectable at 3:1 ratio treatment (Figure 2b). In the presence of *L. innocua*, *L. rhamnosus* showed improved survival (Figure 2c), declining by 2.3-log in the control and 1.7- to 2.3-log in treatments over 96 h.

#### 3.1.2. Effect of Cell-Free Extract of *L. rhamnosus* on Test Organisms

**Figure 3:**
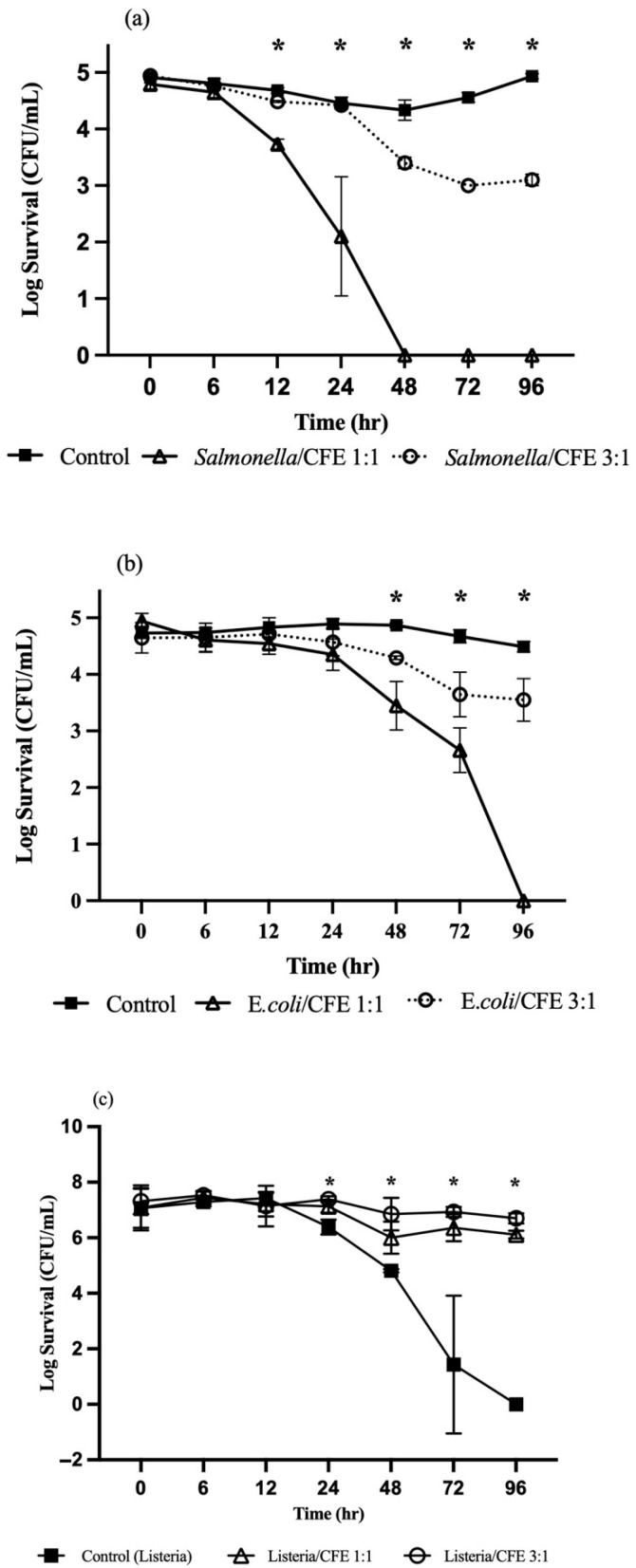
Survival kinetics of *Salmonella* Typhimurium (**a**), *E. coli* O157:H7 (**b**), and *Listeria innocua* (**c**) in *Lactobacillus rhamnosus* cell free extract-treated hydroponic nutrient solution at different proportions. Means with an asterisk indicate significant difference (*p* ≤ 0.05) between treatments.

Figure 3 depicts survival kinetics of pathogens in HNS containing the cell free extract of *L. rhamnosus*. The CFEs significantly reduced (*p* ≤ 0.05) *S.* Typhimurium compared to control (Figure 3a). At a 1:1 ratio, *Salmonella* declined by 2.69 log within 24 h, becoming undetectable thereafter. At 3:1 ratio (i.e., 3 parts *Salmonella* and 1 part CFE), a 1.84 log reduction was observed over 96 h. *E. coli* O157:H7 showed a reductions of 2.28 (1:1) and 1.16 (3:1) log in 96 h (Figure 3b). Interestingly, *L. innocua* survived better in CFE-treated HNS compared to the control (Figure 3c), suggesting potential acid tolerance or proteolytic defense mechanisms.

#### 3.1.3. Effect of Conventional Sanitizers on Test Organisms

**Figure 4:**
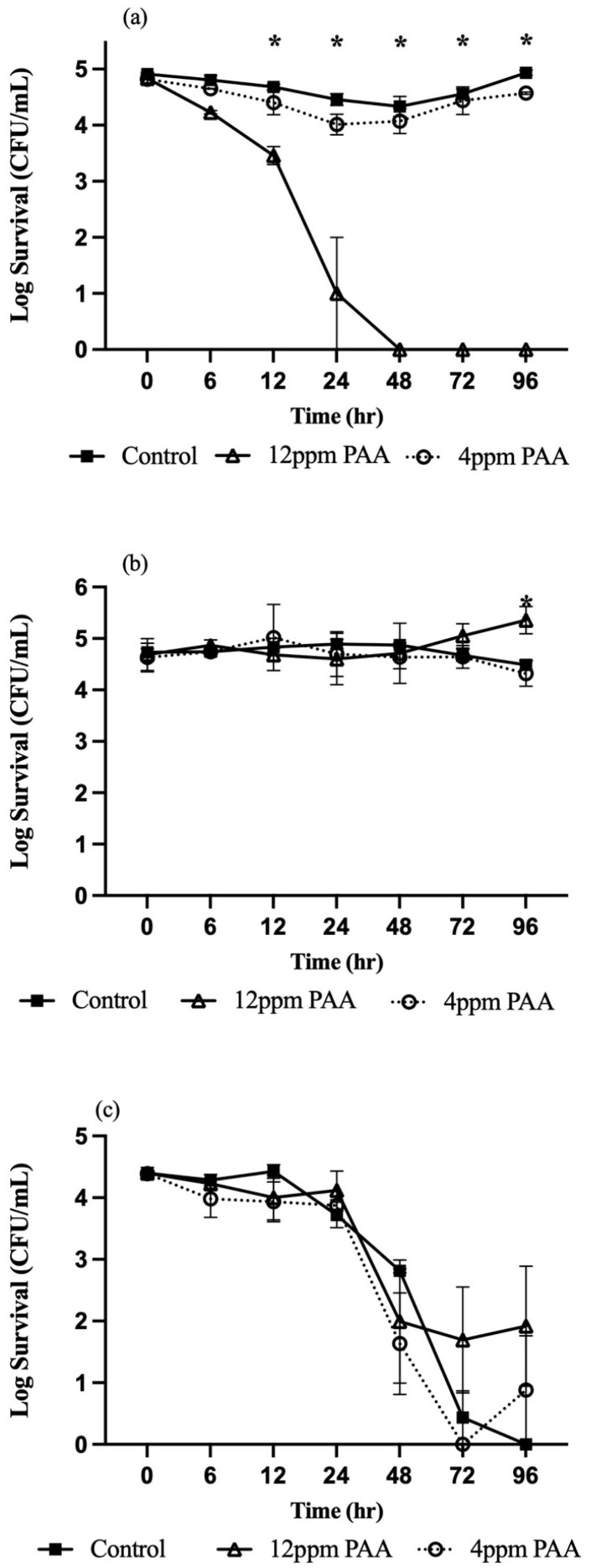
Survival kinetics of *Salmonella* Typhimurium (**a**), *E. coli* O157:H7 (**b**), and *Listeria innocua* (**c**) in peracetic acid (PAA)-treated hydroponic nutrient solutions. Means with an asterisk indicate significant difference (*p* ≤ 0.05) between treatments.

All the tested organisms became undetectable after treatment of HNS with 4 mg/L NaOCl. Increasing PAA concentration from 4 to 12 mg/mL yielded a 3.83 log reduction within 24 h; *Salmonella* was undetectable thereafter (Figure 4a). *E. coli* 0157:H7 was unaffected by 4 and 12 mg/mL PAA (Figure 4b), and *L. innocua* exhibited 2.5 to 4 log reductions independent of PAA concentration compared to control (Figure 4c).

### 3.2. Effect of Antimicrobial Treatments on Plant Growth

**Figure 5:**
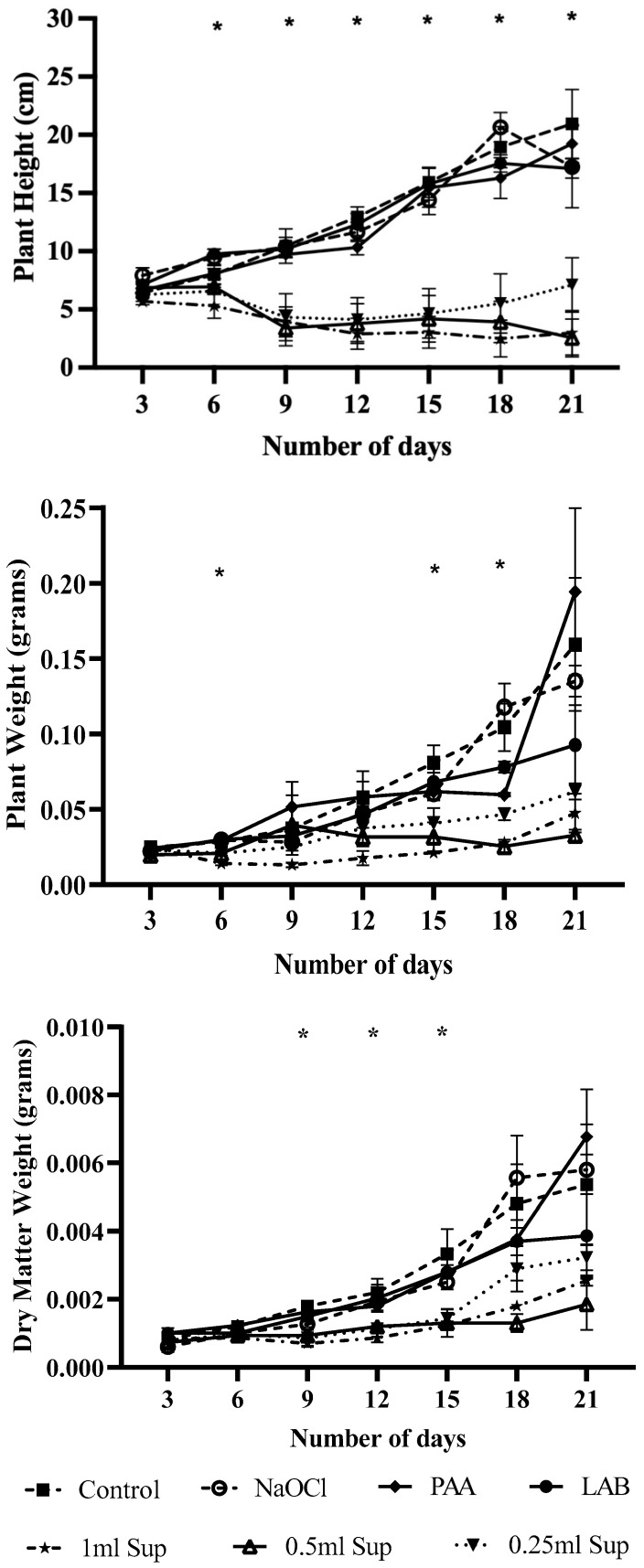
Effect of different treatments on lettuce plant height, weight, and dry matter contents. Means with an asterisk indicate significant difference (*p* ≤ 0.05) between treatments.

Plant growth parameters height, weight, and dry matter under different treatments are shown in Figure 5. Plant height significantly increased in treatments with LAB, PAA, and NaOCl, showing average height gains of 17.12, 14.94, and 15.14 cm, respectively, by day 21, which were comparable to the control. During the early growth period (days 9–12), no marked differences were observed, with 1.52 cm average growth across all treatments (see Supplementary Materials). NaOCl-treated plants achieved the greatest height increase (5.69 cm) on day 15, but declined slightly (1.52 cm) by day 21. Plant weight followed a similar trend, with PAA, NaOCl, and control treatments reaching average weights of 0.19, 0.13, and 0.16 g, respectively, by day 21. LAB and CFE-treated plants showed lower weights overall. Notably, CFE-treated plants experienced weight reductions on days 6, 12, and 18, at certain concentrations, before some recovery occurred later. Dry matter weights also increased ranging from 3.9 to 6.8 mg in the LAB, PAA, NaOCl, and control treatments but remained lower in CFE treatment groups.

### 3.3. Physicochemical Properties of Hydroponic Nutrient Solution

Table 1 shows that pH, EC, TDS, and % Lactic acid content of HNS with CFE over 96 h treatment time when determining the kinetics of microorganisms. No significant difference in the properties of the nutrient solution with cell free extract was observed during this period. Whereas the properties of nutrient solutions while testing plant growth parameters showed fluctuations (see Supplementary Materials). Nutrient availability exhibited minor fluctuations over 21 days. PAA and CFE treatments often had lower pH values (3.98–4.34) compared to LAB, NaOCl, or control treatments (4.56–5.36) on days 0–6, converging after day 12. Electrical conductivity initially varied across treatments (e.g., 133.71 mV in NaOCl vs. 90 mV in control), but stabilized over time.

Total dissolved solids were generally consistent across treatments, with only minor deviations. The 1 mL CFE treatment, however exhibited significantly higher TDS on days 0 and 3 compared to all other groups, while LAB and PAA showed smaller but notable fluctuations (see Supplementary Materials). Nutrient availability showed significant variation primarily during the initial stages of the experiment (days 0, 3, and 9). Specifically, the CFE treatments had significantly higher nutrient availability than the control on days 6 and 9, while LAB, NaOCl, and PAA treatments did not differ significantly from the control. After day 9, no significant differences among treatments were noted; however, within LAB and PAA groups, nutrient availability exhibited significant temporal variations, peaking on days 18 (LAB; 2.18 mS/cm) and 21 (PAA; 2.19 mS/cm).

## 4. Discussion

Hydroponic nutrient solutions, despite their benefits for plant growth, can act as a conduit for microbial contamination. Previous studies demonstrated that foodborne pathogens could attach to plant surfaces or become internalized through contaminated nutrient solutions [7,28,29,30,31,32]. However, limited information is available on sustainable intervention strategies to minimize these risks. This study evaluated the persistence of three major pathogens and/or surrogates *Salmonella enterica* serovar Typhimurium, *Escherichia coli* O157:H7, and *Listeria innocua* in hydroponic nutrient solutions and examined the efficacy of *Lactobacillus rhamnosus* live cells and their cell free extracts as biocontrol interventions. We found that *S.* Typhimurium exhibited only minimal decline (0.57 log CFU/mL) during the test period highlighting its rapid adaptation and survival in nutrient-limited environments in the absence of any antimicrobial interventions. In contrast, a study by Ilic et al. [7] reported a 90% reduction in *S.* Typhimurium levels within 24 h post-inoculation in a hydroponic nutrient solution although it persisted over the 28-day test period. However, other studies suggest that *S.* Typhimurium cells can adapt to their environment quickly even in the presence of other bacteria, leading to a long persistence period [28,33]. Similarly, *E. coli* O157:H7 showed a remarkable resilience, remaining stable in the nutrient solution throughout the study, supporting previous observations of its robust survival in nutrient-deficient aqueous environments [26,34]. *E. coli* O157:H7 was reported to have survived within water samples for up to 91 days [26,33]. It should be noted that nutrient availability is a key determinant of microbial survival in the environment. Soluble carbon sources sustain foodborne pathogens, and in carbon-deficient conditions, *S.* Typhimurium and *E. coli* O157:H7 can utilize nitrate as an alternative electron acceptor [35] during their metabolic activities. Hoaglands Basal 2 nutrient solution used in this study provides nitrate in the form of calcium nitrate and potassium nitrate which could be utilized in carbon deficient HNS environment. In contrast, *L. innocua* exhibited a notable reduction of 4.4-log CFU/mL within 72 h. These findings differ from previous reports that reported longer persistence of *L. innocua* for up to 28 days in stored irrigation water at about 7 °C [36], and *L. monocytogenes* in hydroponic nutrient solution [7]. The differences in the *L. innocua* survival could be attributed to differences in test medium (irrigation water vs. HNS) and test temperatures (7 vs. 23 °C). Furthermore, despite genetic similarities between *L. monocytogenes* and *L. innocua*, slight genomic differences may lead to observable phenotypic differences. *L. innocua* has fewer phosphotransferase systems compared to *L. monocytogenes*, which may affect its fitness in certain environments [37]. These differences in their survival characteristics among different studies are likely attributed to variations in test conditions, such as nutrient solution composition and competitive microbial interactions, as well as strain-specific physiological capabilities.

The limited antagonistic effect of *L. rhamnosus* cells observed in this study contrasts with previous reports demonstrating effective pathogen inhibition in food systems [17,25,38,39]. This discrepancy likely results from the inorganic hydroponic solution used in our experiments, which lacks fermentable substrates essential for lactic acid bacterial metabolism. Such substrate deficiency may have restricted the production of antimicrobial compounds, subsequently causing rapid *L. rhamnosus* cell death [20,40]. Conversely, cell-free extracts of *L. rhamnosus* exhibited significant inhibitory effects against *S.* Typhimurium and *E. coli* O157:H7. This activity may be attributed to metabolites including lactic acid, bacteriocins, or phenolic acids generated during cultivation of *L. rhamnosus* in nutrient-rich media prior to collecting CFE [41,42]. For instance, bacteriocins produced by *L. rhamnosus* have shown antimicrobial efficacy against pathogens such as *S.* Typhimurium, *Staphylococcus* epidermis, *Bacillus cereus*, and *E. coli* [43]. Additionally, chromatographic analysis identified DL-p-hydroxy-phenyllactic acid and ferulic acid in *L. rhamnosus* cultures, both exhibiting considerable inhibitory activity against tested Gram-positive and Gram-negative pathogens [44]. Furthermore, De Keersmaecker et al. [42] reported that heat-stable, low molecular bacteriocins in *L. rhamnosus* CFE are likely responsible for antimicrobial activity against *Salmonella*. Xu et al. [43] also described strong antimicrobial activity of bacteriocins isolated from *L. rhamnosus* against tested Gram-negative bacteria, with weaker or no activity against Gram-positive species, such as *Staphylococcus* aureus. Our findings align closely with these previous observations.

An interesting outcome of our study was the enhanced persistence of *L. innocua* in the presence of *L. rhamnosus* CFE compared to controls. This suggests a potential resilience mechanism in *L. innocua,* possibly involving acid tolerance or bacteriocin resistance [45,46]. Previous studies have indicated that due to inherent acid tolerance and proteolytic enzyme synthesis, the metabolic activities of *L. innocua* may remain unaffected or even enhanced by certain CFEs produced by lactic acid bacteria [18,20,46,47]. Additionally, strains of *L. innocua* have demonstrated resistance to multiple antimicrobials, a trait transferable within *Listeria* species. Kalmokoff et al. [45] identified an inhibitor produced by *L. innocua*, named Listeriocin 743A, which shares similarities with pediocin- like bacteriocins commonly produced by lactic acid bacteria. However, our study was limited to determining only physical properties, pH, and lactic acid concentrations of the tested CFEs. Further in-depth metabolomic profiling studies are necessary to elucidate the precise mechanisms underlying the antimicrobial and supportive growth interactions.

Comparison with conventional chemical treatments indicated that PAA at 12 mg/L effectively reduced *S*. Typhimurium, but lower concentrations (4 mg/mL) showed limited efficacy. *E. coli* O157:H7 was not affected by PAA treatments at tested concentrations. In contrast, CFE was more effective against *E. coli* O157:H7, suggesting its potential for biological treatment in hydroponic systems. This finding reinforces that CFE might offer similar or greater effectiveness against specific pathogens without the potential chemical-related drawbacks. Nevertheless, CFE treatments negatively impacted lettuce growth parameters, likely due to reduced pH and altered nutrient availability, which could limit nutrient uptake and plant health [48].

## 5. Conclusions

This study provides novel insights into the interactions between probiotic *Lactobacillus rhamnosus*, its cell-free extract, and key foodborne pathogens in hydroponic nutrient solutions, while also evaluating the impact of these treatments on plant growth. A key finding is the selective antimicrobial activity of *L. rhamnosus* and its CFE. While live *L. rhamnosus* had limited or no inhibitory effects on *S.* Typhimurium and *E. coli* O157:H7, it significantly mitigated the decline of *L. innocua,* suggesting a potential protective or competitive interaction. In contrast, CFE demonstrated robust antimicrobial activity, particularly against *S.* Typhimurium and *E. coli* O157:H7, with reductions up to 2.69 log CFU/mL, highlighting its potential as a nonviable biocontrol agent. Conventional sanitizers like NaOCl and PAA were highly effective, but their impact on plant growth was comparable to that of LAB treatments suggesting that biocontrol approaches may offer safer, residue-free sustainable alternatives. Future research should explore the mechanistic basis of CFE activity, its spectrum of efficacy, and potential synergistic effects with other biocontrol agents.

## Figures and Tables

**Table 1:** Physico-chemical properties of hydroponic nutrient solution containing cell free extract of *Lactobacillus rhamnosus*.

Time (hr)	Temperature (°C)	pH	Electrical Conductivity (µS/m)	Total Dissolved Solids (ppm)	Lactic Acid (%)
0	23.2 ± 1.4 ^a^	5.55 ± 0.12 ^a^	1328.9 ± 14.8 ^a^	847.5 ± 317.8 ^a^	0.16 ± 0.06 ^a^
6	23.2 ± 0.4 ^a^	5.53 ± 0.08 ^a^	1334.9 ± 21.3 ^a^	849.5 ± 318.8 ^a^	0.15 ± 0.02 ^a^
12	22.7 ± 0.4 ^a^	5.57 ± 0.12 ^a^	1330.0 ± 50.9 ^a^	956.7 ± 20.9 ^a^	0.12 ± 0.00 ^a^
24	22.9 ± 0.5 ^a^	5.51 ± 0.24 ^a^	1331.3 ± 23.2 ^a^	952.2 ± 18.7 ^a^	0.12 ± 0.02 ^a^
48	23.3 ± 0.9 ^a^	5.57 ± 0.11 ^a^	1322.6 ± 35.9 ^a^	941.8 ± 37.9 ^a^	0.12 ± 0.02 ^a^
72	22.8 ± 0.8 ^a^	5.58 ± 0.11 ^a^	1358.7 ± 42.5 ^a^	949.7 ± 30.5 ^a^	0.12 ± 0.03 ^a^
96	22.9 ± 0.3 ^a^	5.58 ± 0.10 ^a^	1341.1 ± 24.1 ^a^	954.8 ± 21.2 ^a^	0.13 ± 0.02 ^a^

Means with same superscript within the same column are not significantly different from each other (*p* ≤ 0.05).

## Data Availability

The original contributions presented in this study are included in the article/supplementary material. Further inquiries can be directed to the corresponding author.

## Supplementary Materials

The following supporting information can be downloaded at: https://www.mdpi.com/article/10.3390/microorganisms13081858/s1, Table S1: Hydroponic nutrient solutions pH (mean and SD); Table S2. Hydroponic nutrient solutions Electrical Conductivity (mean and SD); Table S3. Hydroponic nutrient solutions Total dissolved solids content (mean and SD); Table S4. Nutrient availability (mean and SD).
