# Supplementary material for: Efficacy of Lactobacillus rhamnosus and Its Metabolites to Mitigate the Risk of Foodborne Pathogens in Hydroponic Nutrient Solution
Source: Microorganisms. 2025 Aug 8;13(8):1858. doi: 10.3390/microorganisms13081858 (PMC12388345; doi:10.3390/microorganisms13081858)
Supplement: Supplementary file 1 [file microorganisms-13-01858-s001.zip › microorganisms-3790589-supplementary.pdf]

S1. Hydroponic nutrient solutions pH (mean and SD).

pH measurement							
**Days**	**0.25 ml CFS**	**0.5 ml CFS**	**1 ml CFS**	**Control**	**LAB**	**NaOCl**	**PAA**
0	4.27±0.44^{BCa}	3.95±0.13^{Ca}	3.93±0.21^{Ca}	5.30±0.26^{Aa}	4.79±0.27^{ABa}	4.56±0.30^{ABCa}	4.40±0.24^{BCa}
3	4.16±0.26^{Ba}	3.98±0.21^{Ba}	3.88±0.14^{Ba}	5.34±0.36^{Aa}	5.18±0.50^{Aa}	4.60±0.21^{Aba}	4.21±0.07^{Ba}
6	4.32±0.40 ^{Ba}	4.12±0.46^{Ba}	3.97±0.27^{Ba}	5.36±0.31^{Aa}	5.31±0.37^{Aa}	4.57±0.07^{Aba}	4.34±0.18^{Ba}
9	4.64±0.46^{ABa}	4.31±0.72^{ABa}	3.45±0.99^{Ba}	5.39±0.25^{Aa}	5.29±0.41^{ABa}	4.57±0.08^{Aba}	4.32±0.11^{Aba}
12	5.27±1.07^{ABa}	4.49±0.22^{ABa}	3.97±0.13^{Ba}	5.30±0.62^{Aa}	5.44±0.47^{Aa}	4.76±0.18^{Aba}	4.41±0.25^{Aba}
15	5.05±0.94^{Aa}	4.09±0.34^{Aa}	4.52±1.21^{Aa}	5.04±0.34^{Aa}	5.07±0.29^{Aa}	4.67±0.25^{Aa}	4.39±0.32^{Aa}
18	4.93±0.74^{Aa}	4.75±0.25^{Aa}	3.96±0.23^{Aa}	4.89±0.39^{Aa}	4.94±0.29^{Aa}	4.44±0.16^{Aa}	4.33±0.17^{Aa}
21	5.26±0.70^{Aa}	3.89±0.04^{Aa}	5.27±1.79^{Aa}	4.57±0.99^{Aa}	4.91±0.36^{Aa}	4.69±0.36^{Aa}	4.53±1.04^{Aa}

Observations not connected by the same letter are significantly different. Significant difference between treatment represented with uppercase letters and differences within treatment represented by lowercase letters.

S2. Hydroponic nutrient solutions Electrical Conductivity (mean and SD).

Electrical conductivity (EC) measurement (mV)							
**Days**	**0.25 ml CFS**	**0.5 ml CFS**	**1 ml CFS**	**Control**	**LAB**	**NaOCl**	**PAA**
0	155.47±3.86^{Aa}	160.23±7.22^{Aa}	162.90±8.92^{Aa}	90.00±8.94^{Ba}	111.27±15.88^{Ba}	133.71±3.67^{Aba}	126.93±25.48^{Aba}
3	148.10±14.81^{Aa}	157.43±12.02^{Aa}	163.40±8.08^{Aa}	88.17±23.96^{Ba}	87.63±29.38^{Ba}	121.17±12.91^{Aba}	143.00±5.65^{Aa}
6	142.97±24.75^{Aa}	149.13±26.44^{Aa}	161.70±11.53^{Aa}	85.40±24.28^{BCa}	75.67±17.57^{Ca}	122.73±7.26^{ABCa}	132.80±11.08^{Aba}
9	161.00±1.84^{Aa}	147.40±25.92^{Aa}	160.67±12.82^{Aa}	77.17±13.16^{Aa}	78.53±24.74^{Aa}	121.17±10.9^{Aa}	134.23±9.76^{Aa}
12	113.70±60.76^{Aa}	141.40±2.08^{Aa}	157.83±8.20^{Aa}	80.73±36.08^{Aa}	72.10±27.89^{Aa}	102.00±27.19^{Aa}	131.43±15.30^{Aa}
15	93.87±56.39^{Aa}	148.95±18.74^{Aa}	131.90±59.69^{Aa}	108.33±19.82^{Aa}	93.03±16.92^{Aa}	116.33±25.00^{Aa}	128.23±13.90^{Aa}
18	95.90±53.33^{Aa}	161.60±1.83^{Aa}	106.75±87.33^{Aa}	106.43±22.82^{Aa}	102.20±17.37^{Aaa}	131.53±14.89^{Aa}	138.23±10.50^{Aa}
21	78.73±40.07^{Aa}	163.30±2.26^{Aa}	157.20^{Aa}	123.53±54.50^{Aa}	95.27±16.57^{A}	136.03±16.90^{Aa}	141.80±34.17^{Aa}

Observations not connected by the same letter are significantly different. Significant differences between treatment are represented with uppercase letters and differences within treatment are represented by lowercase letters.

S3. Hydroponic nutrient solutions Total dissolved solids content (mean and SD).

Total dissolved solids measurement (ppt)							
**Days**	**0.25 ml CFS**	**0.5 ml CFS**	**1 ml CFS**	**Control**	**LAB**	**NaOCl**	**PAA**
0	1.155±0.07^{ABa}	1.194±0.01^{ABa}	1.219±0.05^{Aa}	1.084±0.05^{Ba}	1.118±0.01^{ABbc}	1.084±0.05^{Ba}	1.126±0.02^{ABab}
3	1.167±0.04^{ABa}	1.178±0.02^{ABa}	1.213±0.04^{Aa}	1.082±0.06^{Ba}	1.105±0.02 ^{ABcd}	1.081±0.05^{Ba}	1.091±0.03 ^{Bab}
6	1.145±0.06^{Aa}	1.172±0.03^{Aa}	1.174±0.11^{Aa}	1.054±0.01^{Aa}	1.064±0.01^{Ae}	1.076±0.03^{Aa}	1.064±0.02^{Ab}
9	1.195±0.02^{Aa}	1.184±0.02^{Aa}	1.201±0.04^{Aa}	1.076±0.01^{Aa}	1.077±0.01^{Ade}	1.086±0.19^{Aa}	1.096±0.03^{Aab}
12	1.212±0.03^{Aa}	0.9841±0.29^{Aa}	0.980±0.25^{Aa}	1.119±0.05^{Aa}	1.142±0.01^{Aabc}	1.113±0.03^{Aa}	1.062±0.04^{Ab}
15	1.078±0.09^{Aa}	1.068±0.15^{Aa}	1.061±0.09^{Aa}	1.113±0.05^{Aa}	1.120±0.02^{Abc}	1.103±0.03^{Aa}	1.101±0.03^{Aab}
18	0.824±0.23^{Aa}	0.821±0.33^{Aa}	0.949±0.43^{Aa}	1.165±0.03^{Aa}	1.167±0.02^{Aa}	1.101±0.06^{Aa}	1.084±0.04^{Ab}
21	0.993±0.34^{Aa}	0.933±0.39^{Aa}	1.199±0.01^{Aa}	1.133±0.08^{Aa}	1.153±0.01^{Aab}	1.139±0.01^{Aa}	1.173±0.03^{Aa}

Observations not connected by the same letter are significantly different. Significant differences between treatment are represented with uppercase letters and differences within treatment are represented by lowercase letters.

S4. Nutrient availability (mean and SD).

Conditions(mScm^{-1})							
Days	**0.25 ml CFS**	**0.5 ml CFS**	**1 ml CFS**	**Control**	**LAB**	**NaOCl**	**PAA**
0	2.15±0.13^{Aba}	2.23±0.02^{ABa}	2.28±0.09^{Aa}	2.04±0.10^{Ba}	2.09±0.02^{ABbc}	2.03±0.09^{Ba}	2.10±0.03^{ABab}
3	2.18±0.07^{Aba}	2.20±0.04^{Aba}	2.27±0.08^{Aa}	2.02±0.10^{Ba}	2.06±0.04^{Bcd}	2.02±0.10^{Ba}	2.03±0.05^{Bab}
6	2.14±0.11^{Aa}	2.19±0.06^{Aa}	2.19±0.21^{Aa}	1.97±0.02^{Aa}	1.99±.01^{Ad}	2.01±0.05^{Aa}	1.99±0.05^{Ab}
9	2.23±0.04^{Aa}	2.21±0.04^{Aa}	2.29±0.07^{Aa}	2.01±0.02^{Ba}	2.01±0.02^{Bd}	2.03±0.04^{Ba}	2.05±0.04^{Bab}
12	2.26±0.05^{Aa}	1.84±0.54^{Aa}	1.16±0.69^{Aa}	2.09±0.10^{Aa}	2.13±0.01^{Aabc}	2.08±0.06^{Aa}	1.99±0.08^{Ab}
15	2.02±0.17^{Aa}	1.98±0.25^{Aa}	1.98±0.16^{Aa}	2.07±0.10^{Aa}	2.09±0.05^{Abc}	2.06±0.05^{Aa}	2.06±0.06^{Aab}
18	1.69±0.47^{Aa}	1.50±0.55^{Aa}	1.78±0.81^{Aa}	2.18±0.06^{Aa}	2.18±0.03^{Aa}	2.06±0.11^{Aa}	2.03±0.08^{Ab}
21	2.06±0.83^{Aa}	1.74±0.72^{Aa}	2.24±0.01^{Aa}	2.13±0.17^{Aa}	2.15±0.03^{Aab}	1.82±0.55^{Aa}	2.19±0.05^{Aa}

Observations not connected by the same letter are significantly different. Significant difference between treatment are represented with uppercase letters and differences within treatment are represented by lowercase letters.
